# The Role of Light in Enhancing the Nutritional and Antioxidant Qualities of Basil, Mint and Lemon Balm

**DOI:** 10.3390/foods13233954

**Published:** 2024-12-07

**Authors:** Karolina Jakubczyk, Kinga Szymczykowska, Klaudia Melkis, Dominika Maciejewska-Markiewicz, Anna Nowak, Anna Muzykiewicz-Szymańska, Karolina Skonieczna-Żydecka

**Affiliations:** 1 Department of Human Nutrition and Metabolomics, Pomeranian Medical University in Szczecin, Broniewskiego Street 24, 71-460 Szczecin, Poland; kinga.szymczykowska@pum.edu.pl (K.S.); 58147@student.pum.edu.pl (K.M.); dominika.maciejewska@pum.edu.pl (D.M.-M.); 2 Department of Cosmetic and Pharmaceutical Chemistry, Pomeranian Medical University in Szczecin, Powstańców Wielkopolskich Street 72, 70-111 Szczecin, Poland; anna.nowak@pum.edu.pl (A.N.); anna.muzykiewicz@pum.edu.pl (A.M.-S.); 3 Department of Biochemical Research, Pomeranian Medical University in Szczecin, Broniewskiego Street 24, 71-460 Szczecin, Poland; karolina.skonieczna.zydecka@pum.edu.pl

**Keywords:** mint, basil, lemon balm, LED, HPS, antioxidants, polyphenols

## Abstract

Mint (*Mentha* L.), basil, (*Ocimum basilicum*) and Melissa (*Melissa officinalis* L.) are herbaceous plants from the *Lamiaceae* family. They have a wide range of health benefits and flavour properties which are highly valued around the world. Alternative methods of growing plants to minimise greenhouse gas emissions during autumn and winter are being sought in the face of increasing climate change. One way to achieve this is to switch from HPS to LED lighting. LED lighting has a longer lifespan and higher efficiency while using less energy and better matching the colour of the light to the needs of the herbs. This study tested the hypothesis that the type of illumination (solar, HPS, and LED) significantly impacts the antioxidant and nutritional qualities of herbs. The results indicated that LED lighting enhanced biochemical properties, supporting its adoption for sustainable plant cultivation.

## 1. Introduction

In many countries, it is necessary to grow plants in greenhouses during the autumn–winter period. These processes contribute to the emission of greenhouse gases, which is undesirable in times of increasing climate change. Therefore, it is essential to look for methods of growing plants that have the least possible impact on climate change [1]. Until recently, the most popular methods of growing plants in greenhouses were to light them with, for example, incandescent lamps, high-pressure sodium (HPS) lamps, or fluorescent and metal halide lamps. Nowadays, however, LED lamps, which are characterised by the ability to control the amount, frequency, and spectrum of light provided, as well as being energy-efficient, are gaining popularity. This offers a wide range of possibilities, as it allows adaptation to the needs of each crop species and production conditions [2,3]. Lamps of this type do not use a filament or gas discharge and only work through the movement of electrons in a semiconductor material. For this reason, they are considered to be part of “green technology”, as no mercury is released from them, as is the case with conventional illumination systems [4,5]. The wavelength range of LED lamps is 250 nm to 1000 nm. They allow the production of lights of different colours depending on the wavelength: blue (400–500 nm), green (500–600 nm) and red (600–700 nm). It is possible to obtain white colour by using phosphor-coated blue light or by mixing appropriate amounts of light from red, green, and blue diodes [4]. LED lighting has lower production costs and is the most durable and efficient system [1]. Compared to traditional HPS lamps, LED lamps save up to 17% to 47% in electricity costs. Low energy consumption contributes to a reduction in carbon dioxide emissions, which is an additional advantage of solid-state lighting [5,6]. Herbs are also widely grown in greenhouses in addition to fruit and vegetables, which can have a positive impact on their biochemical composition [7].

Basil (*Ocimum basilicum*), which originated in India, is also a herb widely used in Ayurvedic medicine [8]. It belongs to the *Lamiaceae* family and its essential oils are an ingredient in oral hygiene products and dental products, as well as being used in the fragrance and food industries [9]. The main essential oils of basil are eugenol, chavicol, and terpenoids [10]. In addition, basil is a source of phenolic acids, fatty acids (mainly stearic acid, oleic acid, palmitic acid, or linolenic acid), and other components with antioxidant activity, such as quercetin, rutin, or apigenin [11,12]. Thanks to its bioactive compounds, basil has health-promoting properties that find application in the treatment of inflammation, hyperglycaemia, and hyperlipidaemia, alongside antimicrobial and anti-stroke properties. In addition, it has hepatoprotective, cardioprotective, chemopreventive, and anticancer effects [13]. 

Mint (*Mentha L*.) is a plant that also belongs to the *Lamiaceae* family and is originally native to Europe. The mint genus (*Mentha L.*) includes several species of this plant, such as peppermint (*Mentha arvenis L.* lub *Mentha x piperita L.*), black mint (*Mentha vulgaris*), white mint (*Mentha officinalis*), spearmint (*Mentha spicata L.),* and simple mint (*Mentha piperita L.*) [14]. It can be consumed in the form of fresh or dried leaves, or as infusions [14]. Mint is a source of many essential oils (mainly menthol) and health-promoting compounds such as phenolic acids (procatechuic acid, rosmarinic acid, chlorogenic acid) or flavonoids (apigenin, catechin, myricetin) [15]. 

Lemon balm (*Melissa officinalis L.*) also belongs to the *Lamiaceae* family. It is cultivated worldwide for its culinary and health-promoting properties, although it originated in southern Europe and Asia [16]. Most commonly, lemon balm is used as an anti-anxiety agent, and recently, its properties affecting the nervous system and neurocognitive functions have also been studied [17]. In addition, it is used in Asian traditional medicine to treat insomnia and improve memory. The main constituents of lemon balm are hydroxycinnamic acid derivatives, which include caffeic acid, chlorogenic acid, metrilic acid, and rosmarinic acid [18]. 

The type, quality, and intensity of light are crucial to the quality of the plants, not only visually but also in terms of their chemical composition [19]. Light of the right duration can induce synthesis; e.g., polyphenols can be induced in the plant via the phenylpropanoid pathway [6]. Light influences the physiological processes in plants. It is an important factor that affects them not only during growth, but also after harvesting [20]. LED lamps have a positive effect on extending the shelf life of plants after harvesting. It has been shown that postharvest irradiation of plants can extend their shelf life and improve organoleptic properties [21]. Therefore, the aim of this study was to determine what effect the type of lighting (LED, HPS, and solar) has on the selected characteristics of the herbs (mint, lemon balm, and basil). This study hypothesises that the type of illumination (solar, HPS, and LED) significantly affects the antioxidant potential and nutritional qualities of mint, basil, and lemon balm through alterations in their biochemical pathways. By establishing these relationships, this work aims to provide a basis for optimising sustainable and efficient plant cultivation methods.

## 2. Materials and Methods

### 2.1. Plant Material

**Figure 1:**
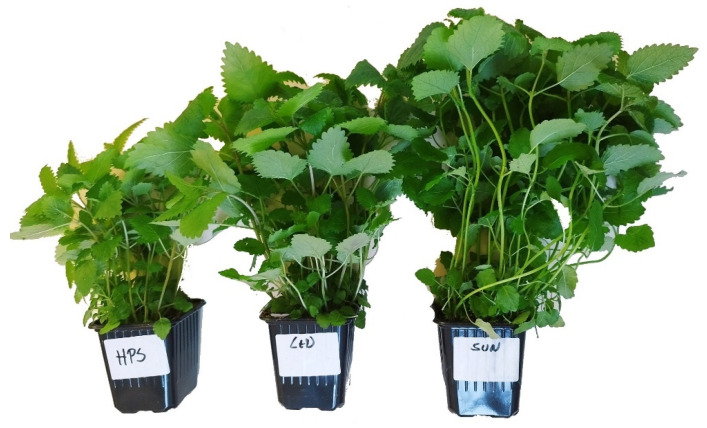
Tested material—mint (*Mentha L.*)—depending on the illumination used (HPS, LED, SUN).

**Figure 2:**
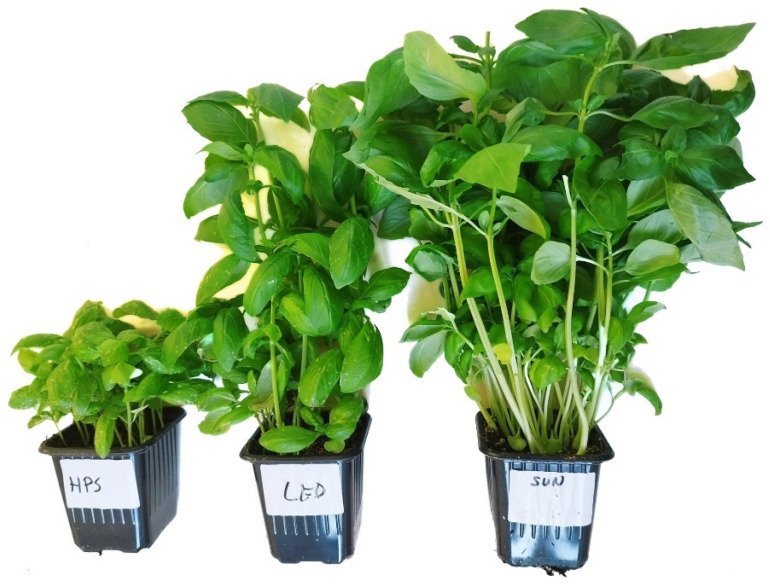
Tested material—basil (*Ocimum basilicum*)—depending on the illumination used (HPS, LED, SUN).

**Figure 3:**
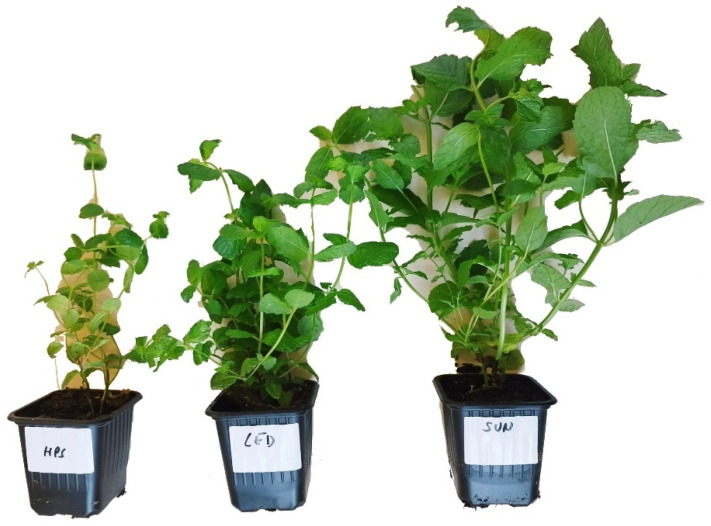
Tested material—lemon balm (*Melissa officinalis L.*)—depending on the illumination used (HPS, LED, SUN).

The test material consisted of 3 herbs—basil, mint, and lemon balm (Table 1). Each herb was subjected to three types of irradiation—LED, HPS, and solar (SUN) (Figure 1, Figure 2 and Figure 3). The average illumination energy at the time of measurement (at the test point) for plants irradiated with LED lamps was 177.56 ± 10.14 µmol/s × m^2^, and for plants irradiated with HPS lamps, this was 93.22 ± 5.95 µmol/s × m^2^.

### 2.2. Herb Cultivation

On the same day, 6 plants of *Mentha* L., *Ocimum basilicum*, and *Melissa officinalis* L. were displayed on a line/table. Mint was the only one to be propagated vegetatively by seedlings. All plants were harvested on the same day and submitted for testing. Accordingly, in each trial, i.e., HPS, LED, and sunlight, there was a control pot filled with peat, without plants, which served as a reference to the pot with plants, in order to be able to estimate the weight of the herb. All test plants were grown under cover (in a greenhouse and in a plastic tunnel) for a uniform climate. In addition, the plants on which the effects of HPS and LED lighting were tested were placed in a purpose-built test room (inside the foil tunnel, for similar temperature, humidity, and CO_2_ content of the air), but without access to any other light source, especially sunlight. The interior of the test room was divided into two halves by a non-permeable curtain. The chambers with HPS as well as LED were equipped with individual sets of fans and thermostats, switching on the fan when the temperature in the chamber exceeded 30 °C. The tested herbs were divided into 3 groups. In each group, the herbs were exposed to a different type of light—sunlight, HPS, or LED. The artificial lighting (LED and HPS) was switched on simultaneously by the controller for a 12 h period from 22:00 to 9:59 on the following day. Temperature, weight, and light spectrum were measured in the morning. The light spectrum was tested with the PG200NSpectral PAR Meter. The measurement was always taken in the same place to increase its reliability. A flood table was used to water the LED- and HPS-exposed herbs. Flood troughs were used on the plant line from the greenhouse. Watering was performed together with fertilisation. Watering in the test room (plants illuminated by HPS and LED) was performed at the same time and with the same nutrient solution as the other plants (illuminated by sunlight). Six plants from each trial were used for the remainder of the experiment.

### 2.3. Preparation of Extracts

Material was taken from 3 different pots, 5 g from each (15 g total), and then crushed; 10 g of crushed material was weighed, transferred to a flask and topped up with solvent to 100 mL. The solvent was water and a mixture of methanol and water (MeOH) prepared in a ratio of 30:70. The extracts were shaken on an absolute shaker (180 RPM) and left overnight in the refrigerator. The extracts were then filtered through a cellulose filter and submitted for analysis.

### 2.4. Antioxidant Activity of Extracts by the DPPH Method

The antioxidant activity of samples was measured with the spectrophotometric method, using synthetic radical DPPH (2.2-diphenyl-1-picrylhydrazyl, Sigma, Poznań, Poland) according to Brand-Williams et al. and Pekkarinen et al. [22,23]. The DPPH stock solution was prepared on the day of analysis by dissolving 0.01183 g of DPPH in 96% ethanol. The DPPH solution was diluted with ethanol to obtain an absorbance of 1.0 ± 0.02 at 518 nm. Then, 3.9 mL of a DPPH ethanolic solution and 0.1 mL of the test sample were added into the vial. The vial contents were thoroughly mixed and incubated in the dark for 30 min at room temperature. The control solution was prepared in the same way but instead of the tested sample, 96% ethanol was added. The spectral absorbance was immediately measured at 518 nm (8453UV, Agilent Technologies, Santa Clara, CA, USA). The results are expressed as % inhibition of DPPH radical. All assays were performed in triplicate [24,25].

### 2.5. Antioxidant Activity of Extracts by the ABTS Method

In total, 0.1 mL of test sample and 2.9 mL of ABTS reagent were introduced into the Falcon-type test tubes. An ABTS stock solution was prepared by mixing 5 mL of 7 mM of an ABTS aqueous solution and 5 mL of 2.45 mM of an potassium persulfate (K_2_S_2_O_8_) aqueous solution, and was incubated in the dark for 12–16 h. The ABTS solution was diluted with ethanol to give an absorbance of 1.00 ± 0.02. Then, 0.1 mL of the tested sample and 2.9 mL of the ABTS reagent were added into the vial. The Falcon was then capped, shaken vigorously, and incubated for 6 min. After the time had elapsed, the absorbance was measured at 734 nm (8453UV, Agilent Technologies, Santa Clara, CA, USA). All assays were performed in triplicate. The results are shown as the % of ABTS radical inhibition [25].

### 2.6. Determination of Reduction Potential of Extracts by the Ferric Ion Reducing Antioxidant Power (FRAP) Method

The FRAP method, used to determine the total reduction potential, is based on the ability of the test sample to reduce Fe^3+^ ions to Fe^2+^ ions. The FRAP unit determines the ability to reduce 1 mole Fe^3+^ to Fe^2+^ [26,27]. A quantity of 3 mL of the FRAP reagent consists of 10 mM tripyridyltriazine (TPTZ) solution, 300 mM acetate buffer (pH = 3.6), and 20 mM FeCl_3_. It was added with the test sample and distilled water into the vial. After mixing, the prepared solution was placed for 5 min at 37 °C. Absorbance at 593 nm was measured (8453UV, Agilent Technologies, Santa Clara, CA, USA). The results are expressed as µM Fe(II)/L. All assays were performed in triplicate [24,25].

### 2.7. Determination of the Total Polyphenol Content (TPC) in Extracts

Determination of the total polyphenol content (TPC) was performed according to ISO 14502-1 and Singleton V.L. and Rossi J.A’s method using Folin–Ciocalteu reagent [28]: 5.0 mL of a Folin–Ciocalteu solution (10%) and 1.0 mL of test sample were added into the vial. The vial contents were thoroughly mixed, and 4.0 mL of 7.5% Na_2_CO_3_ solution was added after 5 min and incubated for 60 min at room temperature. To prepare a reference solution, the same reagents were used, but instead of the tested sample, distilled water was added. Absorbance at 765 nm was measured (8453UV, Agilent Technologies, Santa Clara, CA, USA). All assays were performed in triplicate. The results are shown in mg/L gallic acid [24,25].

### 2.8. Determination of the Total Flavonoid Content (TFC)

Determination of the total flavonoid content was performed according to the methods in Hu et al. [29]. Different concentrations of rutin were used in the plotting of the standard calibration curve; 0.6 mL of a NaNO_2_ solution (5%) and 2.0 mL of test sample were added into the vial. The vial contents were thoroughly mixed and incubated for 6 min, and then 0.5 mL of AlCl_3_ solution (10%) was added. Incubation was repeated under the same conditions. A 4.3% NaOH solution was added, and the 10 mL flask was filled to the line with distilled water. The TFC is expressed as mg of rutin equivalent per 1 L of extract. Absorbance at 510 nm was measured (8453UV, Agilent Technologies, Santa Clara, CA, USA). All assays were performed in triplicate [24,25].

### 2.9. Determination of Water Content of Raw Materials

The water content of the tested herbs was measured using a weighing–drying machine (Radwag model MA 110.R.WH, Radom, Poland). The amount of material and the drying time and temperature were selected according to the manufacturer’s protocols; 1.7 g of raw material was weighted and dried at 110 °C. The trials were repeated twice and the average of the measurements was taken as the final result.

### 2.10. Refractometric Measurements

The contents of magnesium, iron, calcium, vitamin C, glucose, and malic acid were measured using an RQflex^®^ 20 refractometer (Reflequant, Supelco, Inc., Bellefonte, PA, USA); 0.3 mL of the test extract was applied to a test strip and then measured. The detection level of elements (magnesium, iron, and calcium) and malic acid was 5 mg/L, that of vitamin C was 25 mg/L, and that of glucose was 1 mg/L.

### 2.11. Phytochemical Analysis

The following tests were used to detect the presence of phytochemicals: the Salkowski test for triterpenoids, the Fehling test for reducing sugars, the Liebermann–Bourchard reaction for triterpenoids and sterols, the Shinoda reagent test for flavonoids, and a tannin test.

### 2.12. HPLC Analysis

Liquid chromatography (Agilent Technologies 1260 HPLC System, Santa Clara, CA, USA) was used in the order to determinate polyphenol compounds. The column used was a Hypersil Gold (150 × 4.6) with the temperature maintained at 25 ◦C. The detection of phenolic compounds was performed by UV absorption at λ = 278 nm. Each compound was identified based on its retention time and by comparison with standards under the same conditions [30,31]. The mobile phase consisted of 1% aqueous acetic acid solution (A) and 100% MeOH (B). The samples were eluted with the gradient 90% A and 10% B from 0 to 6 min, 84% A and 16% B from 7 to 25 min, 72% A and 28% B from 26 to 37 min, 65% A and 35% B from 38 to 47 min, 50% A and 50% B from 48 to 64 min, and 90% A and 10% B from 65 to 70 min, to restore the initial conditions, before injection of a new sample. The flow rate was 0.8 mL/min, and the injection volume was 30 µL [30,31].

### 2.13. Determination of Chlorophyll and Carotenoids

The chlorophyll and carotenoid content was determined by spectrophotometry by the method described by Liang et al. and Zagórska-Dziok et al. [32,33]. Stock solution of dry extracts at a concentration of 100 µL/mL in 80% acetone was prepared. The absorbance of the solutions was measured at λ = 645 nm, λ = 663 nm, and λ = 470 nm (8453UV, AGILENT TECHNOLOGIES, Santa Clara, CA, USA). The results are expressed as the content of chlorophyll a and b, the total content of chlorophyll (a + b), and the content of carotenoids, calculated in µg/mL. Measurements were carried out in triplicate for each sample of extract [34]. The chlorophyll and carotenoid contents were calculated using the following equations:

Chlorophyll a = 12.7 (A663) − 2.69 (A645),

Chlorophyll b = 22.9 (A645) − 4.68 (A663),

Total chlorophyll (a + b) = 20.2 (A645) + 8.02 (A663),

Carotenoids = ((100 × A470) − (3.27 × Chl a) − (1.04 × Chl b))/229

where

A663—absorbance measured at wavelength 663;
A645—absorbance measured at wavelength 645;
A470—absorbance measured at wavelength 470;
Chl a—content of chlorophyll a [µg/mL];
Chl b—content of chlorophyll b [µg/mL].

### 2.14. Statistical Analysis

Statistical analysis was conducted utilising MedCalc^®^ Statistical Software version 20.218 (MedCalc Software Ltd., Ostend, Belgium; https://www.medcalc.org; accessed on 28 February 2024) and Microsoft Excel 2017. Distributions of values for individual parameters were analysed using the Shapiro–Wilk test [30]. Results are expressed as mean values and standard deviation; however, median values and quartile ranges are used for statistical analyses. The Kruskal–Wallis test was used to evaluate differences between groups due to non-normal distribution, while multivariable regression analysis assessed the interaction effects of illumination and solvent type on biochemical outcomes (e.g., antioxidant capacity and polyphenol content). Significance was determined at *p* ≤ 0.05. This approach ensured robust evaluation of the lighting method’s impact [30].

#### 2.14.1. Multivariable Analysis

A multivariable linear regression analysis was employed to assess the influence of three grouping variables—Name, Lighting_Method, and Solvent—on multiple quantitative response variables. The response variables analysed included the following:

- Antioxidant capacities: ABTS, DPPH, FRAP;
- Polyphenolic contents: Total_polyphenols_content, Total_flavonoids_content;
- Specific biochemical compounds: Apigenin, Kaempferol, Quercetin, Myricetin, Resveratrol, Caffeine, Caffeic_acid, Chlorogenic_acid, Rutin, Four_hydroxybenzoic_acid, Dihydroxybenzoic_acid, Gallic_acid, p_coumaric_acid, Ferulic_acid, Epicatechin_gallate, Sinapic_acid, Ellagic_acid, Two_hydroxycinnaminic_acid.

#### 2.14.2. Regression Analysis

For each response variable, an Ordinary Least Squares (OLS) regression model was fitted. The formula for the OLS regression was constructed to include the main effects of Name, Lighting_method, and Solvent. Models were evaluated for their goodness-of-fit using the R-squared statistic and adjusted R-squared statistic, which indicate the proportion of variance explained by the model. The statistical significance of each predictor was assessed using *p*-values, with a threshold of 0.05 for determining significance.

#### 2.14.3. Handling Multicollinearity

Multicollinearity was assessed by examining the variance inflation factors (VIFs) for the predictor variables. High VIF values indicate a high degree of multicollinearity, which can inflate the standard errors of the coefficients and lead to unreliable statistical inferences.

The statistical analysis in this study was performed using Python, specifically leveraging the following libraries and tools:

- Pandas: Used for data manipulation and preprocessing.
- Statsmodels: Utilised for fitting the Ordinary Least Squares (OLS) regression models and performing the multivariable analysis.
- Jupyter Notebook: Employed as the interactive environment for conducting the analysis and generating the output.

## 3. Results

### 3.1. Antioxidant Activity of the Extracts

The herbs studied were characterised by high antioxidant potential. Analyses were performed using ABTS, DPPH, and FRAP methods (Table 2).

The highest potential measured by the ABTS method was characterised by lemon balm, which had an ABTS radical-reducing capacity of 70.75%. The antioxidant potential of mint was 59.71%, and the antioxidant potential of basil was much lower than the other two herbs—29.85% (Table 2).

For the DPPH method, mint had by far the highest antioxidant potential, with a DPPH radical-reducing capacity of 71.94%. The antioxidant potential of basil, on the other hand, was 16.73% and that of lemon balm was 18.28% (Table 2).

The antioxidant potential measured by the FRAP method for mint and lemon balm was similar. It was 1716.39 µM/L FeII and 1820 µM/L FeII, respectively. However, basil had a significantly lower potential with 715.50 µM/L FeII (Table 2).

In the herbs tested, differences were noted between the solvent used. In mint, methanolic extracts had a significantly higher antioxidant potential for each method used. The median antioxidant potential for the aqueous extracts measured by the ABTS method was 23.19% and that for the methanolic extracts was 97.98%. For the DPPH method, the antioxidant potential of the aqueous extracts was 13.09% and that of the methanolic extracts was 73.79%, while for the FRAP method, the antioxidant potential of the aqueous extracts was 704.06 µM/L FeII and that of the methanolic extracts was 3467.70 µM/L FeII (Table 3).

The methanolic extracts of basil also had a significantly higher antioxidant potential than the aqueous extracts for each of the test methods used. The percentage of ABTS radical inhibition in the aqueous extracts was 11.95% and that in the methanolic extracts was 54.98%. For the DPPH method, the ability was 12.88% in the aqueous extracts and 21.67% in the methanolic extracts. The antioxidant potential measured by the FRAP method for the methanolic extracts was 908.25 µM/L FeII and that for the aqueous extracts was 672.15 µM/L FeII (Table 3).

For basil extracts, the same correlations as for the previous two herbs were recorded. Methanolic extracts had higher antioxidant potential values. They amounted to 84.43% for the ABTS method, 57.00% for the DPPH method, and 2060.10 µM/L FeII for the FRAP method. For aqueous extracts, the values were 30.42% for the ABTS method, 14.96% for the DPPH method, and 1137.5 µM/L FeII for the FRAP method (Table 3).

Statistically significant differences were noted between the irradiation methods used for individual herbs. For mint, differences were recorded for the DPPH method, where the highest antioxidant potential was recorded in SUN herb extracts and was 71.94%. For herb extracts irradiated with LED lamps, the percentage of inhibition of the DPPH radical was 45.17%, which was much higher than for HPS herbs, where the ability was 43.31% (Table 4).

The type of irradiation influenced the antioxidant potential of basil as measured by the ABTS and FRAP methods. For both methods, SUN herbs had the highest antioxidant potential, scoring 42.46% for the ABTS method and 982.71 µM/L FeII for the FRAP method. In contrast, basil irradiated with LED lamps had a significantly higher antioxidant potential compared to herbs irradiated with HPS lamps. For the ABTS method, the LED herb extracts had an antioxidant potential of 34.23% and that for the HPS herb extracts was 26.76%. For the FRAP method, these values were 850.52 µM/L FeII for LED basil extracts and 651.21 µM/L FeII for HPS basil extracts (Table 4).

On the antioxidant potential of lemon balm, the type of illumination had a significant effect for each of the test methods used. For the ABTS and FRAP methods, the highest potential was obtained for SUN lemon balm. The ABTS radical inhibition percentage for it was 99.12%, and by the FRAP method, the value was 5100.70 µM/L FeII. For both methods, LED lemon balm extracts had a significantly higher antioxidant potential compared to HPS lemon balm extracts. The ABTS radical inhibition percentage of the LED extracts was 57.65%, and that of the HPS extracts was 33.89%. For the FRAP method, the antioxidant potential of LED extracts was 1623.60 µM/L FeII, and that of HPS was 997.06 µM/L FeII. For the DPPH method, LED extracts had the highest antioxidant potential with 46.75%, while HPS extracts had 34.07% and SUN extracts had 11.21% (Table 4).

Differences in results between methods used to measure antioxidant potential may be due to certain capabilities and limitations of the methods used. The ABTS and FRAP methods are suitable for the determination of the antioxidant capacity of both hydrophilic and lipophilic compounds [35]. The DPPH radical is only soluble in organic solvents, so the activity of hydrophilic compounds cannot be determined [36].

### 3.2. Total Polyphenol Content (TPC) and Total Flavonoid Content (TFC) in Extracts

Total polyphenol content (TPC) and total flavonoid content (TFC) differed significantly between the herbs tested. The highest TPC value was recorded for lemon balm extracts, in which the content of these compounds was 159.24 mg/L. In mint extracts, the TPC value was 128.43 mg/L, while in basil extracts it was much lower at 45.20 mg/L (Table 5).

In contrast, the highest TFC value was recorded in mint and was 273.62 mg/L. In Melissa, on the other hand, the flavonoid content was 255.03 mg/L. The TFC value in basil extracts was much lower at 77.24 mg/L (Table 5).

The total content of polyphenols and flavonoids in the herbs studied was significantly influenced by the type of solvent used. Significantly higher contents of these compounds were determined in methanolic extracts of mint compared to aqueous extracts. The polyphenol content in the methanolic extracts of mint was 294.57 mg/L, with only 39.32 mg/L in the aqueous extracts. In contrast, the flavonoid content in the methanolic extracts was 683.89 mg/L, while in the aqueous extracts it was 55.42 mg/L (Table 6).

In the basil extracts, TPC and TFC values were also significantly higher in the methanolic extracts. The polyphenol content was 86.05 mg/L in the methanolic extracts and 23.48 mg/L in the aqueous extracts. The flavonoid content, on the other hand, was 88.69 mg/L in the methanol extracts and 28.56 mg/L in the aqueous extracts (Table 6).

The polyphenol content of the lemon balm extracts was significantly higher in the methanolic extracts at 159.24 mg/L, and this value was 136.38 mg/L in the aqueous extracts. On the flavonoid content of the lemon balm extracts, the type of solvent used had no significant effect (Table 6).

For mint and basil extracts, no statistically significant differences were recorded between the types of exposure used. In lemon balm, the highest polyphenol content was determined in SUN plant extracts and was 296.26 mg/L. LED plant extracts contained a significantly higher polyphenol content (93.26 mg/L), compared to HPS plant extracts (56.72 mg/L). Flavonoid content was also highest in SUN lemon balm extracts, the value being 579.90 mg/L. A slightly higher flavonoid content was recorded in HPS plant extracts (155.70 mg/L) compared to LED plant extracts (122.86 mg/L); however, these differences were not statistically significant (Table 7).

### 3.3. Content of Water in Tested Herbs

For water and dry matter content, there were no statistically significant differences between the irradiation methods used on the plants (Table 8).

### 3.4. Phytochemical Analysis

Phytochemical analysis showed that saponins were present in the aqueous extracts of each of the plants tested, with the exception of SUN lemon balm. The methanolic extracts did not show the presence of saponins. Regarding flavonoids, the presence of flavones predominated over chalcones, the presence of which was shown in the aqueous solutions of SUN herbs, LED and HPS basil, and the methanolic extract of HPS mint. Tannin content was detected in each methanolic mint solution, in SUN lemon balm extracts, and in LED lemon balm aqueous extract. No tannins were detected in basil extracts. Reducing sugars were detected in most of the extracts: in the aqueous extracts of mint and basil and the aqueous extracts of SUN and LED lemon balm, and in the methanolic extracts of SUN mint and LED basil. In addition, triterpenoids were detected in every extract tested except in aqueous SUN mint and methanolic SUN basil (Table 9).

### 3.5. Refractometric Measurements

In the tested extracts, the contents of magnesium, iron, glucose, and malic acid were below the detection level. No statistically significant differences were noted between the tested herbs in the case of calcium and vitamin C content (Table 10).

The presence of calcium was not demonstrated in any of the methanol extracts of the herbs studied. In aqueous extracts, the median calcium content was 65 mg/L in mint extracts, 74 mg/L in basil extracts, and 36 mg/L in lemon balm extracts. In contrast to calcium, the content of vitamin C was demonstrated only in the methanol extracts of the herbs studied. In methanol extracts, the median vitamin C content was 32 mg/L in mint extracts, 28 mg/L in basil extracts, and 26 mg/L in lemon balm extracts (Table 11).

The study showed that the type of exposure used had no significant effect on the calcium and vitamin C content in the tested herbal extracts (Table 12).

### 3.6. HPLC Analysis of the Extracts

Statistically significant differences were noted between herbs in the content of polyphenolic compounds. Caffeic acid was not detected in basil, while its content in mint and lemon balm was 0.13 mg/L and 0.44 mg/L, respectively. Concerning ferulic acid, its presence was not detected in mint or lemon balm, while in basil it occurred in the amount of 0.3 mg/L. Differences in the content of kaempferol were noted between basil and lemon balm, in which the content of this compound was 3.62 mg/L and 1.41 mg/L, respectively. The content of quercetin was the highest in mint and amounted to 3.61 mg/L. Significantly lower concentrations of this compound were noted in basil and lemon balm, in which they were 0.13 mg/L and 0.82 mg/L, respectively. Resveratrol was present only in mint, where the median content was 0 mg/L, and the IQR value was 0.23 mg/L. Apigenin was present only in lemon balm, where the median content of this compound was also 0 mg/L, and the IQR value was 0.36 mg/L. Furthermore, the presence of caffeine has only been demonstrated in mint (2.04 mg/L), while it has not been demonstrated in the other herbs. Also, myricetin was found in the highest amount in mint (0.83 mg/L), which was significant compared to other herbs (Table 13).

In the mint extracts studied, the solvent used significantly affected the content of compounds such as chlorogenic acid, kaempferol, resveratrol, and caffeine. Chlorogenic acid and resveratrol were not detected in methanol extracts, while the median of their content in water extracts was 0.27 mg/L and 0.23 mg/L, respectively. The content of kaempferol was higher in methanol extracts at 4.57 mg/L, and in water extracts it was 0.92 mg/L. Similarly, in the case of caffeine, the content in methanol extracts was 4.23 mg/L, and in water extracts it was 0.58 mg/L (Table 14).

In basil extracts, the type of solvent used significantly affected the concentration of chlorogenic acid, ferulic acid, kaempferol, and quercetin. Chlorogenic acid was not present in methanol extracts, and its concentration in aqueous extracts was 0.63 mg/L. Ferulic acid, kaempferol, and quercetin were present in higher concentrations in methanol extracts, and these values were 0.73 mg/L, 4.96 mg/L, and 1.08 mg/L, respectively. In aqueous extracts, the median content of ferulic acid was 0 mg/L, with the IQR value being 0.60 mg/L. The concentration of kaempferol and quercetin in aqueous extracts was 2.62 mg/L and 0.13 mg/L, respectively (Table 14).

In lemon balm extracts, significant differences between solvents were found only in the case of caffeic acid and kaempferol, the content of which in methanol extracts was much higher and amounted to 7.78 mg/L and 2.37 mg/L, respectively. In aqueous extracts, the presence of caffeic acid was not demonstrated, and the amount of kaempferol was 0.48 mg/L (Table 14).

The type of lighting used did not significantly affect the compounds present in the mint extracts tested (Table 15).

In the case of basil, lighting significantly affected the content of ferulic acid, quercetin, and 4-hydroxybenzoic acid. The highest concentration of ferulic acid was recorded for plant extracts exposed to sunlight and its concentration was 2.61 mg/L. In LED plant extracts, it was 0.36 mg/L, and in HPS plant extracts, its presence was not detected. Quercetin was present in the highest amount in LED plant extracts—0.62 mg/L. In SUN and HPS plant extracts, its concentration was 0.50 mg/L and 0.07 mg/L, respectively. 4-hydroxybenzoic acid was not present in LED and HPS plant extracts, and its concentration in SUN plant extracts was 0.08 mg/L (Table 15).

In the tested lemon balm extracts, the type of light exposure significantly affected the content of kaempferol, quercetin, 4-hydroxybenzoic acid, myricetin, and apigenin. Regarding kaempferol, quercetin, and apigenin, their highest concentrations were shown in SUN plant extracts and were 3.48 mg/L, 3.89 mg/L, and 9.59 mg/L, respectively. The lowest kaempferol content was shown in HPS plant extracts—0.28 mg/L. Quercetin was not shown in HPS plant extracts, and the content in LED plant extracts was 0.82 mg/L. Apigenin, on the other hand, was not shown in LED or HPS plant extracts. The presence of 4-hydroxybenzoic acid was detected only in HPS lemon balm extracts and its concentration was 0.07 mg/L, while the content of myricetin was proven only in LED plant extracts, in which it occurred at a concentration of 20.64 mg/L. The sun conditioned the presence of the largest number of compounds, but when comparing artificial lighting methods, LED lamps are more favourable. In particular, the content of quercetin and myricetin in LED plants was even higher than in SUN plants (Table 15).

### 3.7. Chlorophyll and Carotenoid Content

Among the herbs tested, the highest content of total chlorophyll was found in mint and lemon balm, where the concentration of this compound was 5.86 mg/L and 5.58 mg/L, respectively. These values differed significantly from the content of chlorophyll in basil—2.9 mg/L. In each of the herbs, a higher concentration of chlorophyll a was observed compared to chlorophyll b. The content of chlorophyll a in mint and lemon balm was significantly higher than in basil, while the highest content of chlorophyll b was found in mint, where it was significant. When it comes to carotenoids, their highest concentration was determined in mint and basil, where the content of carotenoids was 1.02 mg/L and 0.97 mg/L, respectively. These values differed significantly from lemon balm, where the content of carotenoids was 0.30 mg/L (Table 16).

It was shown that the type of light exposure significantly affects the content of chlorophyll and carotenoids in the tested herbs. In mint, the highest concentration of carotenoids was recorded in HPS plants at 1.63 mg/L, with the lowest in LED plants at 0.85 mg/L. Total chlorophyll and chlorophyll a and b were found in the highest concentration in mint irradiated with sunlight; their content was 8.52 mg/L, 4.94 mg/L, and 3.56 mg/L, respectively. The lowest contents of these compounds were determined in plants irradiated with LED lamps. The concentration of total chlorophyll was 4.94 mg/L, that of chlorophyll a was 2.73 mg/L, and that of chlorophyll b was 2.21 mg/L (Table 17).

For basil, slightly different relationships were shown. The highest concentration of carotenoids was found in SUN plants at 1.12 mg/L, with the lowest in HPS plants—0.86 mg/L. Also, in the case of chlorophyll, the highest concentrations were observed in SUN plants, in which the content of total chlorophyll was 6.63 mg/L, that of chlorophyll a was 3.94 mg/L, and that of chlorophyll b was 2.70 mg/L. The lowest values were characteristic of HPS plants, in which total chlorophyll occurred in the amount of 2.05 mg/L, chlorophyll a occurred in the amount of 1.04 mg/L, and chlorophyll b occurred in the amount of 1.01 mg/L (Table 17).

Lemon balm, in the context of carotenoid content, also shows different dependencies. A significantly higher content of these compounds was found in LED plants and amounted to 0.35 mg/L, with the lowest in SUN plants—0.21 mg/L. However, when it comes to chlorophyll, the highest concentration was also characterised by SUN plants, in which the content of total chlorophyll was 6.45 mg/L, that of chlorophyll a was 3.83 mg/L, and that of chlorophyll b was 2.62 mg/L. The lowest chlorophyll contents in lemon balm were recorded for HPS plants, which contained total chlorophyll in the amount of 4.24 mg/L, chlorophyll a in the amount of 2.42 mg/L, and chlorophyll b in the amount of 1.82 mg/L (Table 17).

### 3.8. Multivariable Regression Analysis

The results from the multivariable regression analyses demonstrate the significant impact of lightening methods and solvents on the biochemical contents of plant extracts (Table 18). Particularly, the use of SUN lighting and the solvent combination water/methanol were consistently associated with higher levels of antioxidant capacities (ABTS and DPPH) and polyphenolic contents. These findings suggest that both light exposure and solvent choice are critical factors in optimising the extraction of beneficial compounds from plants, particularly the following:

ABTS:

The intercept value for ABTS was 14.9772 (*p* = 0.003), indicating the baseline level when other factors are at their reference levels.

Lighting_method had a significant effect: using SUN lighting resulted in a substantial increase in ABTS levels (coefficient = 36.2696, *p* < 0.001).

The solvent water/methanol also significantly increased ABTS levels (coefficient = 50.6467, *p* < 0.001).

DPPH:

The intercept value for DPPH was 4.8963 (*p* = 0.013).

Lighting_method showed a significant effect with SUN lighting (coefficient = 8.9696, *p* = 0.011).

The solvent water/methanol significantly increased DPPH levels (coefficient = 9.1467, *p* = 0.002).

Total polyphenols:

The intercept value for total polyphenols was 30.8762 (*p* < 0.001).

The use of SUN lighting significantly decreased total polyphenol levels (coefficient = −6.8764, *p* = 0.016).

The solvent water/methanol significantly increased total polyphenol levels (coefficient = 9.3467, *p* < 0.001).

Total flavonoids:

The intercept value for total flavonoids was 50.9762 (*p* < 0.001).

SUN lighting significantly decreased total flavonoid levels (coefficient = −15.8764, *p* = 0.020).

The solvent water/methanol significantly increased total flavonoid levels (coefficient = 14.3467, *p* = 0.011).

FRAP:

The intercept value for FRAP was 750.9762 (*p* < 0.001).

Although not all predictors were significant, there was a notable trend, with the solvent water/methanol showing a positive influence on FRAP levels (coefficient = 43.3467, *p* = 0.084), though this did not reach statistical significance.

## 4. Discussion

This study showed that plants under LED lamps have higher fresh and dry mass and a better developed root system than those under HPS lamps. The results supported the hypothesis that light type significantly influences plant biochemistry. Herbs exposed to LED lighting exhibited higher antioxidant potential and total polyphenol content compared to those under HPS lighting, demonstrating the critical role of specific light wavelengths in optimising plant quality. The hypothesis that LED lighting enhances these qualities was validated, aligning with prior research indicating that tailored light spectra influence biochemical pathways. The insights gained from this study are intended to guide sustainable agricultural practices and inform future applied studies on optimising herb cultivation [37,38,39]. This fact is confirmed by this study, in which it was shown that plants irradiated by LED lamps had a slightly higher dry matter content for mint and for basil under LED lighting compared to HPS lamps (Table 8). Regarding lemon balm, the dry matter content was slightly higher for HPS plants. This represents a 3.10% higher dry matter content for mint under LED lights and a 20.59% higher dry matter content for basil under LED lights. The type of illumination also influences the height of the plants, which is significantly higher for LED lamps—188 mm for basil, 120 mm for mint, and 165 mm for lemon balm illuminated by LED lamps compared to, respectively, 63 mm, 110 mm, and 138 mm for plants illuminated by HPS lamps (Table 1). This represents a difference of 185.71% for basil, 9.09% for mint, and 19.57% for lemon balm. Furthermore, the plants illuminated by LED lamps were more vivid in colour and showed no discolouration, unlike those illuminated by HPS lamps. These results are consistent with these obtained by other authors. A study by Aldarkazali et al. investigating the effect of LED lighting on bush basil showed that it increased plants’ fresh and dry weight [40]. In contrast, kale irradiated with UV-A LED light for 5 days prior to harvest was characterised by up to 24.01% and 31.40% higher fresh and dry weights, respectively [41]. According to Marchant et al., red and blue LED light enhanced turmeric sprout growth, with seedlings having higher fresh weight, no signs of etiolation, and higher levels of phytochemicals [42].

In the present study, lemon balm extracts had higher antioxidant potential compared to other extracts (measured by ABTS and FRAP methods). For each of the plants tested and each of the antioxidant potential test methods used, significantly higher results were obtained for the methanol extracts. Abdou found that coriander extracted in water and methanol had a slightly higher antioxidant potential than coriander extracted in water alone. The inhibition values of the DPPH radical were 55.02% for the aqueous methanol extracts and 53.42% for the aqueous extracts of coriander [43]. Lemon balm was found to be characterised by a very high antioxidant potential, and a significantly higher potential was found in the methanol extracts as compared to the aqueous extracts [43].

This study also demonstrated the importance of light exposure on the antioxidant potential of the plants tested. Mint showed a significantly higher antioxidant potential measured by the DPPH method in LED plants compared to HPS plants. Furthermore, LED basil extracts also had a higher antioxidant potential (measured by ABTS and FRAP) compared to HPS plants. The antioxidant potential of lemon balm, on the other hand, was higher for led plants compared to HPS plants for each of the antioxidant potential measurement methods used. Furthermore, the advantage of LED lamps over HPS lamps was shown. Hu et al. demonstrated the beneficial effect of additional UV-A LED light irradiation of kale before harvest. The controls in the study were plants not exposed to LED light. Irradiated kale showed an increased ability to inhibit the DPPH radical by up to 30.87%. Similarly, plants irradiated with LED light 10 days before harvest had a 69.66% higher antioxidant potential as measured by FRAP [41]. LED illumination had a positive effect on the antioxidant potential of coriander, measured by the DPPH method, which increased for blue light and for red light on day 9. The antioxidant potential decreased for plants grown in the dark and irradiated with fluorescent lamps [21]. The same relationships were found for the ABTS and FRAP methods. At the time of harvest, the antioxidant potential was lower then on day 9 [21]. The duration of light exposure during growth is also important for optimal antioxidant characteristics. This was demonstrated by Długosz-Grochowska et al. in a study analysing *V. locusta* grown in winter and autumn. They showed that the antioxidant potential of this plant, measured by the FRAP method, was 203.69 mmol TE/g during winter cultivation in plants exposed to HPS lamps. These values were compared with plants exposed to LED lamps with different percentages of red and blue light. The antioxidant potential was 307.62 mmol TE/g for 90R/10B (90% red light and 10% blue light), 337.35 mmol TE/g for 70R/30B, and 365.11 mmol TE/g for 50R/50B [19]. Higher antioxidant potential in some LED plant extracts may be related to the increased production of bioactive compounds with high antioxidant activity in plants irradiated by LED lamps [44]. Research results presented by other researchers are consistent with those obtained in this study.

Statistically significant differences between studied herbs were also found for both total polyphenol content and total flavonoid content. The highest TPC value was recorded for lemon balm, and the highest TFC value was found in mint. Moreover, significant differences were shown for the extraction methods used. For each herb, both the TFC and the TPC values were significantly higher in the methanolic extracts. The exceptions were lemon balm extracts, where the TFC value was slightly higher in the aqueous extracts, but no statistically significant differences were recorded. The same relationship for polyphenol content as in the present study was found in the study by Wong et al. Their content in the methanolic extracts on a dry weight basis was 152 mg/100 g, and that in the aqueous extracts was 89.3 mg/100 g. In coriander, however, the relationship was reversed, with the polyphenol content in the methanolic extracts being 110 mg/100 g and that in the aqueous extracts being 189 mg/100 g [45]. The use of additional LED lighting in combination with HPS can also have a positive effect on polyphenol levels. In one study, basil irradiated with HPS lamps had a total polyphenol content of 0.95 mg/g, while basil additionally irradiated with LED lamps at a wavelength of 638 nm contained significantly more polyphenols—1.50 mg/g. The same was true for parsley analysed in the same study. Plants exposed to HPS lamps contained 0.62 mg/g of polyphenols, while those exposed to LED lamps contained 1.06 mg/g [46]. In the present study, significant differences between TFC and TPC depending on the type of illumination were noted only for lemon balm. A higher TFC was shown in extracts of LED plants compared to HPS plants, while a higher TFC value was recorded in HPS plants compared to LED. The type of illumination did not significantly affect the flavonoid and polyphenol content of basil and mint. Similar results were obtained in another study in which *Dracocephalum forrestii* grown under blue light combined with red light had the highest phenolic acid content, which is strongly associated with higher antioxidant potential [47]. According to Shoko et al., coriander exposed to LED lights (red and blue light) after harvest had a significantly longer shelf life, increased antioxidant potential, and higher levels of aromatic compounds than plants grown in the dark or exposed to fluorescent lights (white light). On the day of the test, the total polyphenol content was just under 1400 mg/g. Plants grown in the dark or under white light showed a significant decrease in the content of these compounds, to around 950 mg/g and 1100 mg/g, respectively. In plants irradiated with LED lamps, there was an increase in the content of polyphenolic compounds to just over 1500 mg/g under blue light and just under 1500 mg/g under red light [21]. The importance of wavelength on flavonoid content was also demonstrated in the study by Długosz-Grochowska et al. analysing *V. loctusa*. This study was designed to capture different proportions of red and blue light. The content of flavonoid glycosides in plants grown in autumn under LED lamps, with 70% red light and 30% blue light (70R/30B), was 20.54 mg/100 g, and that under 50R/50B was 21.35 mg/100 g. For HPS lamps, the value was 17.05 mg/100 g. However, no statistically significant differences were observed for free flavonoids [19]. The results obtained are consistent with those obtained by other researchers. In the case of polyphenols, including flavonoids, the use of LED lamps is comparable to other methods, such as SUN, which is the most optimal method for plant growth, and HPS, which is a high-energy-consumption method, indicating the beneficial use of LED lamps in plant cultivation.

Phytochemical analysis showed the presence of saponins in every aqueous extract of all herbs studied, except SUN lemon balm, whereas none was detected in the methanolic extracts. Furthermore, the main flavonoid group detected was flavones. Chalcones were only detected in the aqueous SUN extracts of all herbs and in the LED and HPS extracts of basil. Tannins showed their presence in all methanolic extracts of mint, in SUN lemon balm, and in the water extract of LED lemon balm. Reducing sugars were found in most of the extracts, excluding methanolic extracts of LED and HPS mint, methanolic extracts of SUN and HPS basil, methanolic extracts of lemon balm, and water extracts of HPS lemon balm. Triterpenoids, like reducing sugars, were present in most solutions, with the exception of the aqueous extract of SUN mint and the methanolic extract of SUN basil. The nature and ability to dissolve in solvents of different polarity determine the presence of the compounds in question.

Magnesium, iron, glucose, and malic acid were not detected in the extracts. No statistically significant differences in calcium and vitamin C content were noted between the herbs tested. The calcium content in the methanolic extracts was below the detection level, and no vitamin C content was recorded in the aqueous extracts. This may be related to the fact that in the methanolic extracts, vitamin C shows better stability [48]. There was no evidence of any effect of the type of exposure on the content of the tested compounds in each of the herbs.

Analysis by HPLC to identify polyphenolic compounds in the extracts tested showed the presence of chlorogenic acid, caffeic acid, kaempferol, quercetin, resveratrol, caffeine, and myricetin in mint extracts, chlorogenic acid, ferulic acid, quercetin, kaempferol, and 4-hydroxybenzoic acid in basil extracts, and caffeic acid, chlorogenic acid, kaempferol, quercetin, 4-hydroxybenzoic acid, myricetin, and apigenin in lemon balm extracts. Statistically significant differences between herbs were noted for the content of caffeic acid, ferulic acid, kaempferol, quercetin, resveratrol, and apigenin. Statistically significant differences were found in the content of these compounds depending on the extraction method used. In the extracts of each of the herbs tested, kaempferol was the compound found in the highest concentration. It is a compound widely found in plant products and has antioxidant, antimicrobial, cardioprotective, and antiallergic properties [49,50]. In addition, mint has been found to contain an equally high concentration of quercetin, a compound also found widely in herbs. It has antioxidant and antimicrobial properties. It also has the ability to lower blood pressure and may also alleviate allergy symptoms [51,52,53]. In the aqueous extracts of mint, chlorogenic acid and resveratrol were detected, which were not found in the aqueous extracts. Moreover, higher concentrations of compounds such as kaempferol and caffeine were found in methanol extracts of mint compared to aqueous extracts. In basil, on the other hand, differences were found for chlorogenic acid, which was not found in the methanolic extracts, only in the aqueous extracts. Concentrations of ferulic acid, kaempferol, and quercetin were higher in the methanol extracts, compared to the aqueous extracts. In lemon balm, however, the only difference was for caffeic acid, which was detected only in methanolic extracts. The method of irradiation of the plants tested did not significantly affect the content of polyphenolic compounds and caffeine in the mint extracts tested. For lemon balm, the type of illumination used significantly affected the content of kaempferol, quercetin, 4-hydroxybenzoic acid, myricetin, and apigenin. The highest levels of kaempferol and quercetin were found in SUN lemon balm, with the lowest concentrations in HPS extracts. 4-hydroxybenzoic acid was only detected in HPS lemon balm; in addition, myricetin and apigenin were only detected in LED lemon balm. The basil studied by Litvin et al. was characterised by the presence of kaempferol, quercetin, and myricetin. In the case of quercetin, no statistically significant differences were observed between the irradiation methods used. However, the kaempferol content was higher in plants irradiated with HPS lamps, while the myricetin content was significantly higher in plants irradiated with LED lamps [54]. Lettuce plants irradiated with HPS and LED lamps of different wavelengths were studied by Brazaitytė et al. Caffeic acid content was highest in autumn-grown plants irradiated with 660 nm LED lamps (0.151 mg/g), while it was 0.015 mg/g in plants irradiated with HPS lamps. For chlorogenic acid, the highest level was also found in autumn plants irradiated with 455 + 530 nm LED lamps (1.46 mg/g), while plants irradiated with HPS lamps contained less (1.04 mg/g). The highest content of quercetin was 0.180 mg/g in autumn-grown plants exposed to LED lamps at 400 nm wavelength and 0.022 mg/g in plants exposed to HPS lamps. For rutin, the highest content was recorded in the plants grown in autumn and irradiated by LED lamps with a wavelength of 660 nm, and its content was 0.361 mg/g, and in the plants irradiated by HPS lamps, this content was 0.042 mg/g. The highest content of p-coumaric acid was also recorded for plants growing in autumn, illuminated with LED lamps, with a wavelength of 530 nm, and its content was 0.033 mg/g. For plants illuminated with HPS light, these values were 0.008 mg/g [55]. In the study by Długosz-Grochowska et al., the content of some phenolic acids in *V. loctusa* was also examined. The content of chlorogenic acid in plants grown in autumn illuminated with HPS light was 57.47 mg/100 g; in plants illuminated with LED lamps with 90% red light and 10% blue light (90R/10B), it was 66 mg/100 g, whereas for 70R/30B plants it was 72.15 mg/L and for 50R/50B plants it was 70.81 mg/100 g [19]. The content of p-coumaric acid in plants grown in winter illuminated with HPS lamps was 0.97 mg/100 g, and in plants illuminated with LED lamps of different wavelengths, these values were in the range of 1.08–1.17 mg/100 g. For ferulic acid, statistically significant differences were also noted in *V. loctusa* growing in winter. Its content in plants illuminated with HPS lamps was 0.34 mg/100 g. Significantly higher values were noted for LED lamps with 100R, 90R/10B, and 50R/50B lights, in which the concentration of ferulic acid was 0.43 mg/100 g, 0.45 mg/100 g, and 0.42 mg/100 g, respectively [19]. In this study, the results obtained were consistent with the results of other researchers. Similar relationships were also demonstrated regarding the type of light used. Herbs are a valuable source of polyphenolic substances with many health-promoting properties.

The quality of light emitted plays an important role in photosynthesis, affecting how light is absorbed by chlorophyll. For example, red light is important for shoot/stem elongation, phytochrome reactions, changes in stomatal conductance, and plant anatomy [56]. Blue light, on the other hand, plays an important role in chlorophyll biosynthesis, the opening of stomata, enzyme synthesis, chloroplast maturation, and photosynthesis. Blue light has a positive and coordinated effect on both genomes, nuclear and plastid genomes, and chloroplast development in plant cells [56,57,58]. In this study, the highest total chlorophyll content was found in lemon balm and mint. The highest content of carotenoids was found in mint. This study showed that the type of lighting used significantly influenced the assimilation pigment content of the plants analysed. In all plants tested, the highest chlorophyll contents were found in extracts of SUN plants. In mint extracts, however, higher total chlorophyll contents were determined in extracts of HPS plants compared to LED plants. For basil and lemon balm extracts, on the other hand, the relationships were different, as significantly higher chlorophyll contents were shown in LED compared to HPS extracts. For carotenoids, their content in mint extracts was lower for LED plants compared to HPS plants. In basil and lemon balm extracts, significantly higher concentrations of carotenoids were recorded in LED plant extracts compared to HPS plants. In previous studies conducted, exposure to blue-white LED light increased chlorophyll and carotenoid levels in broccoli [59]. One of the reasons for the inhibition of chlorophyll degradation in leaves under red LED irradiation is the suppression of the expression of genes that degrade this pigment, such as chlorophyllases II (BoCLH_2_), chlorophyllases III (BoCLH_3_), and pheophorbide-oxygenase (BoPAO) [60]. In the study by Litvin et al., chlorophyll a content in basil did not change significantly with the applied light intensity. These conclusions are different from the present study, because the total chlorophyll content in basil was significantly higher in LED plants compared to HPS. Litvin et al. showed, however, that the chlorophyll b content was significantly higher in plants illuminated with low-blue-light-ratio LED lamps compared to HPS lamps, which is consistent with the results of the present study [54]. This is confirmed by other studies in which the LED light used also increased the assimilatory pigment content of chlorophyll in the leaves of plants such as *Chlorella ellipsoidea* [58], *Brassica campestris* [57], *Musa acuminata* [56], and *Braccoli* [60].

## 5. Conclusions

In summary, the tested herbs are rich sources of antioxidants, including polyphenolic compounds such as caffeic acid, chlorogenic acid, and catechins. The given properties and phytochemical composition are influenced by the type of solvent used. An increased content of bioactive compounds (polyphenols, flavonoids, quercetin, kaempferol, caffeine) and enhanced antioxidant potential were demonstrated in the methanol extracts. In addition, the use of LED lamps has many benefits, among which are lower energy consumption, better developed plants, and more intense colour compared to HPS lamps. The use of LED lamps also had a positive effect on the increase in antioxidant potential and the content of polyphenol compounds (total polyphenols, quercetin, myricetin) compared to HPS lamps. The chemical composition of plants grown under LED lamps was also similar to those grown under sunlight, which is an additional advantage of using this type of lighting to grow plants in autumn and winter. Overall, the demonstrated benefits of LED lighting—such as enhanced antioxidant activity and reduced energy consumption—suggest its potential for broad adoption in commercial herb cultivation. Future research should explore scaling these findings to industrial applications, considering cost-effectiveness and environmental sustainability.

## Figures and Tables

**Table 1:** Characteristics of the test material.

Herb	Green Weight of the Adult Plant	Growth Time (from Sowing to Harvesting)	Shelf Life (How Long it Lasts from Harvesting Without Being Watered)	Height Measured at the Edge of the Pot—Initial Measurement [mm]			Height Measured at the Edge of the Pot—End Measurement [mm]		
	[g]	Days	Days	SUN *n* = 6	LED *n* = 6	HPS *n* = 6	SUN *n* = 6	LED *n* = 6	HPS *n* = 6
Mint (*Mentha L.*)	20	Not applicable	4–5	5	5	5	235	120	110
Basil (*Ocimum basilicum*)	40	29	6–7	1	1	1	250	188	63
Lemon balm (*Melissa officinalis L.)*	28	26	6–7	-	-	-	205	165	138

**Table 2:** Antioxidant activity (ABTS, DPPH, FRAP) of mint, basil, and lemon balm extracts.

	Mint		Basil		Lemon Balm	
	Median	IQR	Median	IQR	Median	IQR
ABTS [%]	59.71 ^b^	74.78	29.85 ^a,c^	43.03	70.75 ^b^	68.23
DPPH [%]	71.94 ^b^	60.63	16.73 ^a^	8.80	18.28	43.61
FRAP [µM/L FeII]	1716.39 ^b^	2763.64	715.50 ^a,c^	257.52	1820 ^b^	3647.40

Different letters indicate that the median/IQR values between columns in the same row are significantly different (*p* < 0.05). IQR: (interquartile range).

**Table 3:** Antioxidant activity (ABTS, DPPH, FRAP) of mint, basil, and lemon balm extracts divided by the type of solvent.

	Mint			
	Water		MeOH	
	Median	IQR	Median	IQR
ABTS [%]	23.19 *	5.27	97.98 *	3.72
DPPH [%]	13.09 *	3.06	73.79 *	2.40
FRAP [µM/L FeII]	704.06 *	202.11	3467.70 *	944.75
	Basil			
ABTS [%]	11.95 *	6.05	54.98 *	22.22
DPPH [%]	12.88 *	2.57	21.67 *	25.14
FRAP [µM/L FeII]	672.15 *	92.17	908.25 *	643.83
	Lemon balm			
ABTS [%]	30.42 *	83.52	84.43 *	42.25
DPPH [%]	14.96 *	5.93	57.00 *	74.79
FRAP [µM/L FeII]	1137.5 *	4921.34	2060.10 *	3123.85

* Statistically significant difference between types of solvent (*p* < 0.05). IQR: (interquartile range).

**Table 4:** Antioxidant activity (ABTS, DPPH, FRAP) of mint, basil, and lemon balm extracts divided by the lighting method.

	Mint					
	SUN		LED		HPS	
	Median	IQR	Median	IQR	Median	IQR
ABTS [%]	64.46	70.94	56.65	67.20	59.15	78.71
DPPH [%]	71.94 ^b^	59.06	45.17 ^a,c^	59.11	43.31 ^b^	61.35
FRAP [µM/L FeII]	2107.47	2808.94	1716.39	1664.30	1993.20	2811.77
	Basil					
ABTS [%]	42.46 ^c^	60.07	34.23 ^c^	43.03	26.76 ^a,b^	37.63
DPPH [%]	27.92	35.97	17.82	7.94	15.42	4.30
FRAP [µM/L FeII]	982.71^c^	675.96	850.52 ^c^	147.65	651.21 ^a,b^	4.96
	Lemon balm					
ABTS [%]	99.12 ^b,c^	0.83	57.65 ^a,c^	54.02	33.89 ^a,b^	41.43
DPPH [%]	11.21 ^b,c^	20.01	46.75 ^a^	63.14	34.07 ^a^	43.61
FRAP [µM/L FeII]	5100.70 ^b,c^	666.60	1623.60 ^a,c^	922.60	997.05 ^a,b^	1090.74

Different letters indicate that the median/IQR values between columns in the same row are significantly different. (*p* < 0.05). IQR: (interquartile range).

**Table 5:** Total content of polyphenols and total content of flavonoids in mint, basil, and lemon balm extracts.

	Mint		Basil		Lemon Balm	
	Median	IQR	Median	IQR	Median	IQR
TPC [mg/L]	128.43 ^b^	255.25	45.20 ^a,c^	62.57	159.24 ^b^	261.95
TFC [mg/L]	273.62 ^b^	628.48	77.24 ^a,c^	60.14	255.03 ^b^	416.88

Results are presented as median and IQR. Different letters (a–c) represent different herbs: a—mint; b—basil; c—lemon balm. Different letters indicate that the median/IQR values between columns in the same row are significantly different (*p* < 0.05). IQR: (interquartile range).

**Table 6:** Total content of polyphenols and total content of flavonoids in mint, basil, and lemon balm extracts divided by the type of solvent.

	Mint			
	Water		MeOH	
	Median	IQR	Median	IQR
TPC [mg/L]	39.32 *	8.06	294.57 *	79.84
TFC [mg/L]	55.42 *	17.18	683.89 *	210.75
	Basil			
TPC [mg/L]	23.48 *	6.57	86.05 *	49.27
TFC [mg/L]	28.56 *	12.56	88.69 *	80.50
	Lemon balm			
TPC [mg/L]	136.38 *	253.84	159.24 *	218.52
TFC [mg/L]	477.87	547.67	245.97	494.92

* Statistically significant difference between types of solvent (*p* < 0.05). IQR: (interquartile range).

**Table 7:** Total content of polyphenols and total content of flavonoids in mint, basil, and lemon balm extracts divided by the lighting method.

	Mint					
	SUN		LED		HPS	
	Median	IQR	Median	IQR	Median	IQR
TPC [mg/L]	166.39	255.25	128.43	176.22	163.71	266.28
TFC [mg/L]	377.05	652.61	273.62	414.79	362.80	636.73
	Basil					
TPC [mg/L]	69.34	95.42	56.45	57.19	42.14	42.86
TFC [mg/L]	99.97	171.37	77.24	45.88	55.83	72.39
	Lemon balm					
TPC [mg/L]	296.26 ^b,c^	86.38	93.26 ^a,c^	126.04	56.72 ^a,b^	100.54
TFC [mg/L]	579.70 ^b,c^	151.76	122.86 ^a^	233.25	155.70 ^a^	96.23

Different letters indicate that the median/IQR values between columns in the same row are significantly different (*p* < 0.05). IQR: (interquartile range).

**Table 8:** Water and dry matter content of the test material.

	Mint					
	SUN		LED		HPS	
	Median	IQR	Median	IQR	Median	IQR
Water [%]	87.28	0.31	88.04	0.62	88.40	0.03
Dry matter [%]	12.72	0.31	11.96	0.62	11.60	0.03
	Basil					
Water [%]	86.78	2.71	91.80	1.33	93.20	1.07
Dry matter [%]	13.22	2.71	8.20	1.33	6.80	1.07
	Lemon balm					
Water [%]	85.76	1.54	87.17	0.64	86.71	3.83
Dry matter [%]	14.24	1.54	12.83	0.64	13.29	3.83

IQR: (interquartile range).

**Table 9:** Phytochemical analysis of extracts.

Material	Lighting	Extract	Saponins	Flavonoids	Tannins	Reducing Sugars	Triterpenoids
Mint	SUN	water	+	Chalkones	-	+	-
		MeOH	-	Flavones	+	+	+
	LED	water	+	Flavones	-	+	+
		MeOH	-	Flavones	+	-	+
	HPS	water	+	Flavones	-	+	+
		MeOH	-	Chalkones	+	-	+
Basil	SUN	water	+	Chalkones	-	+	+
		MeOH	-	Flavones	-	-	-
	LED	water	+	Chalkones	-	+	+
		MeOH	-	Flavones	-	+	+
	HPS	water	+	Chalkones	-	+	+
		MeOH	-	Chalkones	-	-	+
Lemon balm	SUN	water	-	Flavones	+	+	+
		MeOH	-	Flavones	+	-	+
	LED	water	+	Flavones	+	+	+
		MeOH	-	Flavones	-	-	+
	HPS	water	+	Chalkones	-	-	+
		MeOH	-	Chalkones	-	-	+

-: not detected; +: detected.

**Table 10:** Content of Mg, Fe, Ca, vitamin C, glucose, and mallic acid in examined extracts depending on the type of herb.

	Mint		Basil		Lemon Balm	
	Median	IQR	Median	IQR	Median	IQR
Ca [mg/L]	65	47.25	74	21.25	36	6
Vitamin C [mg/L]	32	2.25	28	3.75	26	0
Glucose [mg/L]	<1	-	<1	-	<1	-
Mg [mg/L]	<5	-	<5	-	<5	-
Fe [mg/L]	<5	-	<5	-	<5	-
Mallic acid [mg/L]	<5	-	<5	-	<5	-

Detection levels of the elements (magnesium, iron, and calcium) and malic acid were 5 mg/L, that of vitamin C was 25 mg/L, and that of glucose was 1 mg/L. IQR: (interquartile range). -: No detected.

**Table 11:** Content of Mg, Fe, Ca, vitamin C, glucose, and mallic acid in extracts depending on the type of the solvent.

	Mint			
	Water		MeOH	
	Median	IQR	Median	IQR
Ca [mg/L]	65	47.25	<5	-
Vitamin C [mg/L]	<25	-	32	2.25
Glucose [mg/L]	<1	-	<1	-
Mg [mg/L]	<5	-	<5	-
Fe [mg/L]	<5	-	<5	-
Mallic acid [mg/L]	<5	-	<5	-
	Basil			
Ca [mg/L]	74	21.75	<5	-
Vitamin C [mg/L]	<25	-	28	3.75
Glucose [mg/L]	<1	-	<1	-
Mg [mg/L]	<5	-	<5	-
Fe [mg/L]	<5	-	<5	-
Mallic acid [mg/L]	<5	-	<5	-
	Lemon balm			
Ca [mg/L]	36	6	<5	-
Vitamin C [mg/L]	<25	-	26	-
Glucose [mg/L]	<1	-	<1	-
Mg [mg/L]	<5	-	<5	-
Fe [mg/L]	<5	-	<5	-
Mallic acid [mg/L]	<5	-	<5	-

Statistically significant difference between type of solvent (*p* < 0.05). Detection levels of the elements (magnesium, iron, and calcium) and malic acid were 5 mg/L, that of vitamin C was 25 mg/L, and that of glucose was 1 mg/L. IQR: (interquartile range), -: no detected.

**Table 12:** Content of Mg, Fe, Ca, vitamin C, glucose, and mallic acid in mint, basil, and lemon balm extracts divided by the lighting method.

	Mint					
	SUN		LED		HPS	
	Median	IQR	Median	IQR	Median	IQR
Ca [mg/L]	65	0	57	0	120	0
Vitamin C [mg/L]	32	0	29	0	32	0
Glucose [mg/L]	<1	-	<1	-	<1	-
Mg [mg/L]	<5	-	<5	-	<5	-
Fe [mg/L]	<5	-	<5	-	<5	-
Mallic acid [mg/L]	<5	-	<5	-	<5	-
	Basil					
Ca [mg/L]	102	0	74	0	73	0
Vitamin C [mg/L]	28	0	28	0	33	0
Glucose [mg/L]	<1	-	<1	-	<1	-
Mg [mg/L]	<5	-	<5	-	<5	-
Fe [mg/L]	<5	-	<5	-	<5	-
Mallic acid [mg/L]	<5	-	<5	-	<5	-
	Lemon balm					
Ca [mg/L]	<5	-	39	0	33	0
Vitamin C [mg/L]	<25	-	26	0	<25	-
Glucose [mg/L]	<1	-	<1	-	<1	-
Mg [mg/L]	<5	-	<5	-	<5	-
Fe [mg/L]	<5	-	<5	-	<5	-
Mallic acid [mg/L]	<5	-	<5	-	<5	-

Detection levels of the elements (magnesium, iron, and calcium) and malic acid were 5 mg/L, that of vitamin C was 25 mg/L, and that of glucose was 1 mg/L. IQR: (interquartile range), -: no detected.

**Table 13:** Content of polyphenolic compounds and caffeine in analysed extracts of mint, basil, and lemon balm.

	Mint		Basil		Lemon Balm	
	Median	IQR	Median	IQR	Median	IQR
Caffeic acid [mg/L]	0.13 ^b^	0.57	ND ^a,c^	ND	0.44 ^b^	7.87
Chlorogenic acid [mg/L]	0	0.27	0	0.63	0.30	0.93
Ferulic acid [mg/L]	ND ^b^	ND	0.3 ^a,c^	0.73	ND ^b^	ND
Kaempferol [mg/L]	2.17	3.65	3.62 ^c^	2.34	1.41 ^b^	2.91
Quercetin [mg/L]	3.61 ^b,c^	3.19	0.13 ^a^	1.05	0.82 ^a^	1.95
Resveratrol [mg/L]	0 ^b,c^	0.23	ND ^a^	ND	ND ^a^	ND
Rutin [mg/L]	ND	ND	ND	ND	ND	ND
Gallic acid [mg/L]	ND	ND	ND	ND	ND	ND
Dihydroxybenzoic acid [mg/L]	ND	ND	ND	ND	ND	ND
4-hydroxybenzoic acid [mg/L]	ND	ND	0	0 **	0	0 **
Ellagic acid [mg/L]	ND	ND	ND	ND	ND	ND
Caffeine [mg/L]	2.04 ^b,c^	3.65	ND ^a^	ND	ND ^a^	ND
p-coumaric acid [mg/L]	ND	ND	ND	ND	ND	ND
Epicatechin gallate [mg/L]	ND	ND	ND	ND	ND	ND
Sinapic acid [mg/L]	ND	ND	ND	ND	ND	ND
2-hydroxybenzoic acid [mg/L]	ND	ND	ND	ND	ND	ND
Myricetin [mg/L]	0.83 ^b,c^	2.78	ND ^a^	ND	0 ^a^	0 **
Apigenin [mg/L]	ND ^c^	ND	ND ^c^	ND	0 ^a,b^	0.36

Different letters indicate that the median/IQR values between columns in the same row are significantly different (*p* < 0.05). ** Minimum and maximum (min–max) values of the detected polyphenolic compounds: Basil: 4-hydroxybenzoic acid—0–0.16 mg/L. Lemon balm: 4-hydroxybenzoic acid—0–0.18 mg/L; myricetin—0.00–42.10 mg/L. ND—not detected, IQR: (interquartile range).

**Table 14:** Content of polyphenolic compounds and caffeine in mint, basil, and lemon balm extracts divided by the type of solvent.

	Mint			
	Water		MeOH	
	Median	IQR	Median	IQR
Caffeic acid [mg/L]	0.54	0.46	0	0.54
Chlorogenic acid [mg/L]	0.27 *	0.32	ND *	ND
Ferulic acid [mg/L]	ND	ND	ND	ND
Kaempferol [mg/L]	0.92 *	1.29	4.57 *	2.48
Quercetin [mg/L]	3.13	2.20	4.94	5.52
Resveratrol [mg/L]	0.23 *	0.37	ND *	ND
Rutin [mg/L]	ND	ND	ND	ND
Gallic acid [mg/L]	ND	ND	ND	ND
Dihydroxybenzoic acid [mg/L]	ND	ND	ND	ND
4-hydroxybenzoic acid [mg/L]	ND	ND	ND	ND
Ellagic acid [mg/L]	ND	ND	ND	ND
Caffeine [mg/L]	0.58 *	2.84	4.23 *	4.49
p-coumaric acid [mg/L]	ND	ND	ND	ND
Epicatechin gallate [mg/L]	ND	ND	ND	ND
Sinapic acid [mg/L]	ND	ND	ND	ND
2-hydroxybenzoic acid [mg/L]	ND	ND	ND	ND
Myricetin [mg/L]	0.13	2.74	1.52	10.52
Apigenin [mg/L]	ND	ND	ND	ND
	Basil			
Caffeic acid [mg/L]	ND	ND	ND	ND
Chlorogenic acid [mg/L]	0.63 *	0.64	ND *	ND
Ferulic acid [mg/L]	0 *	0.60	0.73 *	4.62
Kaempferol [mg/L]	2.62 *	1.18	4.96 *	3.63
Quercetin [mg/L]	0.13 *	0.13	1.08 *	1.08
Resveratrol [mg/L]	ND	ND	ND	ND
Rutin [mg/L]	ND	ND	ND	ND
Gallic acid [mg/L]	ND	ND	ND	ND
Dihydroxybenzoic acid [mg/L]	ND	ND	ND	ND
4-hydroxybenzoic acid [mg/L]	0	0.15	ND	ND
Ellagic acid [mg/L]	ND	ND	ND	ND
Caffeine [mg/L]	ND	ND	ND	ND
p-coumaric acid [mg/L]	ND	ND	ND	ND
Epicatechin gallate [mg/L]	ND	ND	ND	ND
Sinapic acid [mg/L]	ND	ND	ND	ND
2-hydroxybenzoic acid [mg/L]	ND	ND	ND	ND
Myricetin [mg/L]	ND	ND	ND	ND
Apigenin [mg/L]	ND	ND	ND	ND
	Lemon balm			
Caffeic acid [mg/L]	ND *	ND	7.87 *	39.38
Chlorogenic acid [mg/L]	0.62	0.87	0	12.33
Ferulic acid [mg/L]	ND	ND	ND	ND
Kaempferol [mg/L]	0.48 *	3.34	2.37 *	2.93
Quercetin [mg/L]	0	1.92	1.63	5.95
Resveratrol [mg/L]	ND	ND	ND	ND
Rutin [mg/L]	ND	ND	ND	ND
Gallic acid [mg/L]	ND	ND	ND	ND
Dihydroxybenzoic acid [mg/L]	ND	ND	ND	ND
4-hydroxybenzoic acid [mg/L]	0	0.15	ND	ND
Ellagic acid [mg/L]	ND	ND	ND	ND
Caffeine [mg/L]	ND	ND	ND	ND
p-coumaric acid [mg/L]	ND	ND	ND	ND
Epicatechin gallate [mg/L]	ND	ND	ND	ND
Sinapic acid [mg/L]	ND	ND	ND	ND
2-hydroxybenzoic acid [mg/L]	ND	ND	ND	ND
Myricetin [mg/L]	ND	ND	0	41.27
Apigenin [mg/L]	0	0.36	0	19.29

Results are presented as median and IQR. * Statistically significant difference between type of solvent (*p* < 0.05). IQR: (interquartile range). ND—not detected.

**Table 15:** Content of polyphenolic compounds and caffeine in mint, basil, and lemon balm extracts divided by the lighting method.

	Mint					
	SUN		LED		HPS	
	Median	IQR	Median	IQR	Median	IQR
Caffeic acid [mg/L]	0.57	0.06	0	0.61	0	0.12
Chlorogenic acid [mg/L]	0.16	0.33	0.13	0.27	ND	ND
Ferulic acid [mg/L]	ND	ND	ND	ND	ND	ND
Kaempferol [mg/L]	3.13	2.45	1.56	1.60	2.82	4.39
Quercetin [mg/L]	3.82	1.81	3.70	4.21	2.07	4.40
Resveratrol [mg/L]	ND	ND	0.18	0.40	0.11	0.23
Rutin [mg/L]	ND	ND	ND	ND	ND	ND
Gallic acid [mg/L]	ND	ND	ND	ND	ND	ND
Dihydroxybenzoic acid [mg/L]	ND	ND	ND	ND	ND	ND
4-hydroxybenzoic acid [mg/L]	ND	ND	ND	ND	ND	ND
Ellagic acid [mg/L]	ND	ND	ND	ND	ND	ND
Caffeine [mg/L]	0.86	0.53	2.87	5.55	3.67	1.25
p-coumaric acid [mg/L]	ND	ND	ND	ND	ND	ND
Epicatechin gallate [mg/L]	ND	ND	ND	ND	ND	ND
Sinapic acid [mg/L]	ND	ND	ND	ND	ND	ND
2-hydroxybenzoic acid [mg/L]	ND	ND	ND	ND	ND	ND
Myricetin [mg/L]	2.14	1.26	0.06	0.13	5.26	10.52
Apigenin [mg/L]	ND	ND	ND	ND	ND	ND
	Basil					
Caffeic acid [mg/L]	ND	ND	ND	ND	ND	ND
Chlorogenic acid [mg/L]	0.32	0.64	0.31	0.64	ND	ND
Ferulic acid [mg/L]	2.61 ^b,c^	4.07	0.36 ^a,c^	0.73	ND ^a,b^	ND
Kaempferol [mg/L]	3.00	3.04	5.59	5.61	3.76	2.34
Quercetin [mg/L]	0.50 ^b^	1.08	0.62 ^a,c^	0.99	0.07 ^b^	0.10
Resveratrol [mg/L]	ND	ND	ND	ND	ND	ND
Rutin [mg/L]	ND	ND	ND	ND	ND	ND
Gallic acid [mg/L]	ND	ND	ND	ND	ND	ND
Dihydroxybenzoic acid [mg/L]	ND	ND	ND	ND	ND	ND
4-hydroxybenzoic acid [mg/L]	0.08 ^b,c^	0.16	ND ^a^	ND	ND ^a^	ND
Ellagic acid [mg/L]	ND	ND	ND	ND	ND	ND
Caffeine [mg/L]	ND	ND	ND	ND	ND	ND
p-coumaric acid [mg/L]	ND	ND	ND	ND	ND	ND
Epicatechin gallate [mg/L]	ND	ND	ND	ND	ND	ND
Sinapic acid [mg/L]	ND	ND	ND	ND	ND	ND
2-hydroxybenzoic acid [mg/L]	ND	ND	ND	ND	ND	ND
Myricetin [mg/L]	ND	ND	ND	ND	ND	ND
Apigenin [mg/L]	ND	ND	ND	ND	ND	ND
	Lemon balm					
Caffeic acid [mg/L]	19.69	43.31	3.58	7.87	0.44	0.96
Chlorogenic acid [mg/L]	6.01	13.23	0.42	0.93	0.30	0.62
Ferulic acid [mg/L]	ND	ND	ND	ND	ND	ND
Kaempferol [mg/L]	3.48 ^b,c^	0.51	1.33 ^a^	1.89	0.28 ^a^	0.62
Quercetin [mg/L]	3.89 ^b,c^	4.44	0.82 ^a,c^	1.63	ND ^a,b^	ND
Resveratrol [mg/L]	ND	ND	ND	ND	ND	ND
Rutin [mg/L]	ND	ND	ND	ND	ND	ND
Gallic acid [mg/L]	ND	ND	ND	ND	ND	ND
Dihydroxybenzoic acid [mg/L]	ND	ND	ND	ND	ND	ND
4-hydroxybenzoic acid [mg/L]	ND ^c^	ND	ND ^c^	ND	0.07 ^a,b^	0.16
Ellagic acid [mg/L]	ND	ND	ND	ND	ND	ND
Caffeine [mg/L]	ND	ND	ND	ND	ND	ND
p-coumaric acid [mg/L]	ND	ND	ND	ND	ND	ND
Epicatechin gallate [mg/L]	ND	ND	ND	ND	ND	ND
Sinapic acid [mg/L]	ND	ND	ND	ND	ND	ND
2-hydroxybenzoic acid [mg/L]	ND	ND	ND	ND	ND	ND
Myricetin [mg/L]	ND ^b^	ND	20.64 ^a,c^	41.27	ND ^b^	ND
Apigenin [mg/L]	9.59 ^b,c^	20.34	ND ^a^	ND	ND ^a^	ND

Different letters indicate that the median/IQR values between columns in the same row are significantly different. (*p* < 0.05). ND—not detected, IQR: (interquartile range).

**Table 16:** Chlorophyll and carotenoid content in mint, basil, and lemon balm.

	Mint		Basil		Lemon Balm	
	Median	IQR	Median	IQR	Median	IQR
Carotenoids [mg/L]	1.02 ^c^	0.76	0.97 ^c^	0.27	0.30 ^a,b^	0.13
Chlorophyll a [mg/L]	3.03 ^b^	2.21	1.56 ^a,c^	2.89	3.30 ^b^	1.39
Chlorophyll b [mg/L]	2.83 ^b,c^	1.32	1.35 ^a,c^	1.67	2.28 ^a,b^	0.76
Total chlorophyll [mg/L]	5.86 ^b^	3.53	2.9 ^a,c^	4.56	5.58 ^b^	2.15

Different letters indicate that the median/IQR values between columns in the same row are significantly different. (*p* < 0.05). IQR: (interquartile range).

**Table 17:** Chlorophyll content of mint, basil, and lemon balm divided according to the type of irradiation used.

	Mint					
	SUN		LED		HPS	
	Median	IQR	Median	IQR	Median	IQR
Carotenoids [mg/L]	1.02 ^b,c^	0.01	0.85 ^a,c^	0.03	1.63 ^a,b^	0.01
Chlorophyll a [mg/L]	4.94 ^b,c^	0.01	2.73 ^a,c^	0.03	3.03 ^a,b^	0.01
Chlorophyll b [mg/L]	3.56 ^b,c^	0.03	2.21 ^a,c^	0.08	2.83 ^a,b^	0.01
Total chlorophyll [mg/L]	8.52 ^b,c^	0.03	4.94 ^a,c^	0.1	5.86 ^a,b^	0.02
	Basil					
Carotenoids [mg/L]	1.12 ^b,c^	0.08	0.97 ^a^	0.09	0.86 ^a^	0.002
Chlorophyll a [mg/L]	3.94 ^b,c^	1.19	1.56 ^a,c^	0.38	1.04 ^a,b^	0.004
Chlorophyll b [mg/L]	2.70 ^b,c^	0.68	1.35 ^a,c^	0.24	1.01 ^a,b^	0.01
Total chlorophyll [mg/L]	6.63 ^b,c^	1.86	2.91 ^a,c^	0.62	2.05 ^a,b^	0.01
	Lemon balm					
Carotenoids [mg/L]	0.21 ^b,c^	0.01	0.35 ^a,c^	0.01	0.30 ^a,b^	0.01
Chlorophyll a [mg/L]	3.83 ^b,c^	0.02	3.30 ^a,c^	0.04	2.42 ^a,b^	0.01
Chlorophyll b [mg/L]	2.62 ^b,c^	0.04	2.28 ^a,c^	0.05	1.82 ^a,b^	0.02
Total chlorophyll [mg/L]	6.45 ^b,c^	0.06	5.58 ^a,c^	0.09	4.24 ^a,b^	0.03

Different letters indicate that the median/IQR values between columns in the same row are significantly different (*p* < 0.05). IQR: (interquartile range).

**Table 18:** Multivariable regression analysis. Significant predictors (*p* < 0.05) are highlighted with asterisks (**). Only key response variables with meaningful results are presented. The table does not include variables that resulted in non-informative or invalid model summaries.

Response Variable	Predictor	Coefficient	Standard Error	t-Value	*p*-Value
**ABTS**	Intercept	14.9772	4.632	3.233	0.003
	Name [T.LEMONBALM]	7.7914	3.926	1.985	0.057
	Name [T.MINT]	7.1857	4.302	1.670	0.106
	Lighting_method [T.LED]	11.6733	8.265	1.412	0.169
	Lighting_method [T.SUN]	36.2696	8.265	4.389	<0.001 **
	Solvent [T.WATER/METHANOL]	50.6467	6.810	7.437	<0.001 **
**DPPH**	Intercept	4.8963	1.837	2.665	0.013
	Name [T.LEMONBALM]	1.8714	1.557	1.202	0.239
	Name [T.MINT]	2.4668	1.707	1.445	0.158
	Lighting_method [T.LED]	3.4837	3.280	1.062	0.297
	Lighting_method [T.SUN]	8.9696	3.280	2.734	0.011
	Solvent [T.WATER/METHANOL]	9.1467	2.703	3.384	0.002
**Total Polyphenols**	Intercept	30.8762	1.500	20.584	<0.001 **
	Name [T.LEMONBALM]	−1.1771	1.271	−0.926	0.363
	Name [T.MINT]	0.8214	1.394	0.589	0.560
	Lighting_method [T.LED]	−4.6737	2.681	−1.744	0.091
	Lighting_method [T.SUN]	−6.8764	2.681	−2.564	0.016
	Solvent [T.WATER/METHANOL]	9.3467	2.211	4.226	<0.001 **
**Total Flavonoids**	Intercept	50.9762	3.610	14.121	<0.001 **
	Name [T.LEMONBALM]	−2.1471	3.059	−0.702	0.489
	Name [T.MINT]	1.6214	3.354	0.484	0.632
	Lighting_method [T.LED]	−9.6737	6.442	−1.502	0.144
	Lighting_method [T.SUN]	−15.8764	6.442	−2.465	0.020
	Solvent [T.WATER/METHANOL]	14.3467	5.311	2.702	0.011
**FRAP**	Intercept	750.9762	16.430	45.709	<0.001 **
	Name [T.LEMONBALM]	−12.1471	13.927	−0.872	0.390
	Name [T.MINT]	18.6214	15.275	1.219	0.233
	Lighting_method [T.LED]	−30.6737	29.330	−1.046	0.305
	Lighting_method [T.SUN]	−48.8764	29.330	−1.666	0.106
	Solvent [T.WATER/METHANOL]	43.3467	24.180	1.793	0.084

## Data Availability

The original contributions presented in the study are included in the article, further inquiries can be directed to the corresponding author.
